# Test–Retest Reliability and Sex-Dependent Responses for Physiological and Perceptual Variables at Sub-Maximal Thresholds

**DOI:** 10.3390/jfmk10040448

**Published:** 2025-11-19

**Authors:** Erik R. Snell, Pasquale J. Succi, Clara J. Mitchinson, Brian Benitez, Minyoung Kwak, Alaina N. Kuhn, Haley C. Bergstrom

**Affiliations:** 1 Department of Kinesiology and Health Promotion, University of Kentucky, Lexington, KY 40506, USA; esn227@uky.edu (E.R.S.);; 2 Center for Natural and Behavioral Sciences, Exercise Science, Chestnut Hill College, Philadelphia, PA 11918, USA; 3 Department of Health Sciences, Stetson University, DeLand, FL 32723, USA

**Keywords:** exercise prescription, cardiorespiratory fitness, fatigue, reliability

## Abstract

**Background:** Fatigue thresholds such as the gas exchange threshold (GET) and respiratory compensation point (RCP) describe unique physiological responses. This study investigated the reliability of, and sex-dependent responses for, the GET and RCP across absolute, relative, and normalized expressions of volume of oxygen consumption ($\dot{V}$O_2_), power output (PO), heart rate (HR), and rating of perceived exertion (RPE). **Methods**: A test–retest graded exercise test (GXT) protocol was conducted on healthy, recreationally trained males (n = 9) and females (n = 9) to determine the GET and RCP ($\dot{V}$O_2_). Linear regression was used to identify the PO, HR, and RPE at the GET and RCP. Separate 2 [test (1, 2)] × 2 [sex (male, female)] mixed-model analysis of variance (ANOVA) examined systematic error across test and sex (*p* > 0.05). Separate reliability analyses were conducted for each variable for males and females using intraclass correlation coefficients (ICC_2,1_), minimal differences (MDs), standard errors of measure (SEMs), and coefficients of variation (CVs). **Results**: Absolute and relative expression of PO and $\dot{V}$O_2_ at the GET and RCP reflected “excellent” relative reliability (R = 0.816–0.978) across sex. Absolute and normalized expression of HR and RPE at the GET and RCP demonstrated “good” to “poor” relative reliability (R= −0.093–0.886) across sex. The SEMs and MDs were relatively small with CVs at or below 10% across thresholds. Absolute PO and $\dot{V}$O_2_ for the GET and RCP were greater for males than females, while females demonstrated greater normalized RCP as well as absolute and normalized HR and RPE at the RCP. **Conclusions**: Although the relative reliability for HR and RPE at the GET and RCP was, in some cases, limited, these variables demonstrated acceptable absolute reliability and, therefore, have applicability to monitor and prescribe exercise intensities. Current exercise prescription techniques may neglect the unique sex-dependent perceptual and cardiovascular responses at the GET and RCP.

## 1. Introduction

The American College of Sports Medicine exercise prescription guidelines provide light, moderate, and vigorous exercise intensity recommendations based on measures of rating of perceived exertion (RPE), percentage of heart rate maximum, percentage of oxygen consumption maximum, percentage of heart rate reserve, and oxygen consumption ( $\dot{V}$O_2_) reserve [1]. A limitation of prescribing exercise relative to $\dot{V}$O_2_ and HR maximum or reserve is the assumption that the physiological responses at these relative intensities are homogenous across all individuals. Previous work, however, has demonstrated that relative intensities (maximum or reserve) do not elicit the same physiological response across individuals [2]. Alternatively, fatigue thresholds, which reflect transition points of physiological and perceptual responses across exercise intensities, are individually derived and serve as useful tools in exercise prescription [3,4,5,6]. Fatigue thresholds or “submaximal anchors”, such as the gas exchange threshold (GET), ventilatory threshold (VT), respiratory compensation point (RCP), and critical power (CP), have been proposed [7] to separate the three exercise intensity domains (moderate, heavy, and severe) [8]. These domains are described by specific patterns of responses for $\dot{V}$O_2_ and blood lactate as indicators of aerobic and anaerobic contributions, respectively.

The GET, determined as the point of departure from linearity in the $\dot{V}$CO_2_ versus $\dot{V}$O_2_ relationship [5] and has been proposed as the demarcation point of the moderate and heavy exercise intensity domains [7]. Thus, the GET identifies the transition point where anaerobic energy systems supplement aerobic energy reconstitution. The RCP, defined by increased ventilation ( $\dot{V}$_E_) relative to $\dot{V}$CO_2_ [5], likely reflects the point where hydrogen ion production exceeds buffering capacity. Theoretically, the RCP identifies the highest work rate where metabolic responses ( $\dot{V}$O_2_ and blood lactate) reach a steady state. Similarly, CP has been proposed to reflect the highest intensity where a metabolic steady state is obtained [9]. Although subject to debate [10,11,12,13], the RCP and CP occur around the transition from the heavy to severe exercise intensity domains.

Previous work [14,15,16,17,18,19] has examined the reliability of gas exchange and ventilatory thresholds (i.e., GET, RCP, and ventilatory thresholds 1 [VT_1_] and 2 [VT_2_]). These studies have demonstrated strong reliability for the absolute (power output (PO), L·min^−1^, running speed) [14,15,16,17] and relative (mL·kg^−1^·min^−1^ or W·kg^−1^) expressions of these thresholds [14,17,18,19], with limited evaluation [14,17] of reliability for values normalized to maximal responses (i.e., $\dot{V}$O_2max_). In addition, the evaluation of reliability has been predominantly limited to males [14,15,16,18] or mixed samples of males and females [17]. Evidence suggests, however, that unique sex-dependent responses in PO, $\dot{V}$O_2_, HR, and RPE may occur during submaximal exercise [19,20,21,22]. Sex-dependent responses in absolute expressions of PO and $\dot{V}$O_2_ are well-documented, and the superior responses for males likely reflect their greater body size, muscle mass, and hemoglobin concentrations compared to females [23,24,25]. These differences are typically, but not always, accounted for by normalization to maximal capacities [26]. Similarly, RPE was shown to differ between sexes at a fixed relative $\dot{V}$O_2_ (mL·kg^−1^·min^−1^) during cycling trials [26]. However, when $\dot{V}$O_2_ responses were normalized to maximal capacity, RPE displayed no significant differences between males and females [26]. Recent evidence also suggests there may be differences in absolute and normalized HR responses at submaximal thresholds. Specifically, females have demonstrated higher absolute and normalized HR responses at VT_1_ [22], which occurs at a similar intensity as the GET. Evidence suggests [27] that females can have up to a 23% lower stroke volume compared to males, requiring higher heart rates to meet the demands of cardiac output. Given these potential differences in submaximal responses, the reliability of submaximal thresholds across absolute, relative, and normalized expressions for $\dot{V}$O_2_ and PO, as well as the associated HR and RPE, should be established for males and females.

Although PO and $\dot{V}$O_2_ serve as established reliable measures of exercise prescription, they have limited application to the general population. Individuals in the general population often do not have access to research or training facilities with metabolic carts, portable gas exchange analyzers, or cycling power pedals. Therefore, although reliable, exercise prescription using PO and $\dot{V}$O_2_ is an inaccessible option for many individuals. The use of HR and RPE serves as a more accessible option for exercise prescription; however, the reliability of these parameters associated with thresholds such as the GET and RCP has not been fully established in males and females. As described, HR is often prescribed based on an individual’s HR maximum or reserve [28] and can lack consistency in targeting desired metabolic responses amongst individuals [7]. In addition, Borg’s 15-point RPE scale [29] not only serves as a measure of perceptual effort but also as a method of prescribing [30] and anchoring exercise intensity [31,32]. Rather than using an arbitrary HR percentage or RPE, sensitivity could be improved when using the HR and RPE associated with fatigue thresholds (GET and RCP) that define responses across the exercise intensity domains. No previous studies, however, have simultaneously examined the HR and RPE associated with the GET and RCP in males and females during cycle ergometry exercise. Therefore, the purpose of this study was to investigate the reliability of, and sex-dependent responses for, the GET and RCP across absolute, relative, and normalized expressions of PO, $\dot{V}$O_2_, HR, and RPE.

Based on previous evidence, it was hypothesized that the PO and $\dot{V}$O_2_ associated with the GET and RCP would demonstrate strong relative and absolute reliabilities [14,15,16,17,18,19]. Few studies have investigated the reliability of the HR and RPE associated with the GET or RCP [17,18,33,34], but evidence [17,18,33,34] suggests that HR and RPE demonstrate strong relative reliability. Thus, we anticipated similar strong reliability across expressions in this study. We also hypothesized that males would demonstrate greater absolute PO and $\dot{V}$O_2_ values than females at the GET and RCP, but when expressed in relative and normalized terms, sex differences would no longer be present. Finally, although some evidence suggests that sex differences may occur at submaximal intensities [22,26], we hypothesized that there would be no sex differences in the HR or RPE associated with the GET and RCP due to their expression relative to individual thresholds.

## 2. Materials and Methods

### 2.1. Experimental Design

This study used a test–retest model to determine the reliability of two submaximal thresholds (GET and RCP), as well as the HR and RPE associated with these thresholds. The study design comprised three total visits. Visit one served as a familiarization with the GXT protocol. This provided the participants with a greater understanding of the testing procedures and physiological effort required for the test; it also served to familiarize the subjects with the RPE scale. Visits two and three were the experimental visits, where the subjects completed a GXT on a cycle ergometer for the determination of the GET and RCP, as well as the HR and RPE associated with each threshold.

### 2.2. Subjects

This study included 18 moderately trained, recreationally active males (n = 9; Mean ± SD, 24 ± 4 years, 180 ± 9 cm, 80.0 ± 9.3 kg) and females (n = 9; Mean ± SD, 22 ± 2 years, 167 ± 3 cm, 58.3 ± 7.4 kg). Given the test–retest nature of this study, the sample size was determined based on the effect size needed to demonstrate a large effect (d = 0.8–1.2) if differences existed between test days. A power analysis (G*Power 3.1.9.6) indicated that a standardized effect size of 1.2 for meaningful differences in fatigue thresholds and a power of 0.80 at *p* < 0.05 required 8 subjects. This sample size was consistent with previous work examining the reliability of physiological and perceptual fatigue thresholds [19]. The subjects in this study were part of an overlapping sample from previous studies that examined the test–retest reliability of peak values from the GXT [35,36,37,38], but the submaximal values examined in this study were not previously reported. Individuals were eligible for inclusion if they performed structured moderate intensity physical activity for 30 min (min) 3 or more d·wk^−1^ for 3 months prior to participating [1] (pp. 38). In addition, individuals were eligible if they had no known cardiovascular, metabolic, or musculoskeletal disease or disorders. The subjects were asked to maintain their current level of physical activity but to abstain from high-intensity exercise at least 24 h prior to their testing session and from caffeine consumption 4 h before their testing session. There was no control for the menstrual cycle in this study. The test–retest nature of this study, however, was likely short enough (2–5 days between visits) to mitigate the influence of physiological changes across phases of the menstrual cycle [39]. All subjects completed a health history form and signed a written informed consent document prior to beginning this study. This study was approved by the University’s Institutional Review Board for Human Subjects (IRB#64999) and conducted in accordance with the ethical principles of the Declaration of Helsinki.

### 2.3. Experimental Procedures

#### 2.3.1. Graded Exercise Test

Each subject completed a familiarization and two experimental GXTs on a calibrated cycle ergometer, with each test separated by ≥48 h. The first visit served as a familiarization test, where the subjects completed the GXT to exhaustion as they would on the second and third visits, so that subjects understood the effort required for each visit. The familiarization also ensured that the subjects were comfortable with the mouthpiece and cycle ergometer, so that their responses for the subsequent tests reflected their true physiological limits and were not constrained by the novelty of the task. This also served as a familiarization with the Borg 6-20 RPE scale [29]. Visits two and three consisted of a step incremental GXT protocol to investigate the test–retest determination of submaximal thresholds (GET and RCP) and the HR and RPE associated with each threshold.

Prior to testing, gas analyzers were calibrated to room air and gases of their known concentrations (16% O_2_, 4% CO_2_; acceptable tolerance limit ≤ 0.1%). Additionally, the flow meter was calibrated with a 3 L syringe (Hans Rudolph, Series 5530, 3 Liter Calibration Syringe, Shawnee, KS, USA) with an acceptable tolerance limit of ±1%. Expired gas samples were collected and analyzed every 20 s using a calibrated TrueOne 2400 metabolic cart (Parvo Medics, Sandy, UT, USA). Each subject was then fitted with a mouthpiece attached to a headset (Hans Rudolph 2700 breathing valve, Kansas City, MO, USA), a nose clip, and a chest heart rate monitor (Polar Heart Watch system; Polar Electro Inc., Lake Success, NY, USA) used to record 20 s HR averages during the GXT. Each subject completed a 4 min warmup at a power output of 50 W. Upon completion of the warmup, the subjects received 1 min of passive recovery. Each GXT followed a step incremental protocol starting at a power output of 50 W, with a subsequent increase of 30 W every 2 min. This protocol has been established as a reliable and valid method of achieving $\dot{V}$O_2max_ in recreationally trained males and females [35,36]. The RPE was recorded upon completion of each incremental stage to examine the perceptual effort of exercise using Borg’s 6-20 scale. PO continued to increase until the subjects reached volitional fatigue, marked by a decrease in cadence below 70 revs⋅min^−1^ for 10 s despite verbal encouragement. The peak power output (PPO) was recorded as the stage with the highest PO prior to termination of the test. The highest 20 s $\dot{V}$O_2_ and HR value recorded during the test was defined as the $\dot{V}$O_2peak_ and HR_peak_, respectively, while the highest recorded RPE was considered RPE_peak_.

#### 2.3.2. Determination of the GET and RCP

The GET was determined through visual inspection as the point of departure from linearity of the $\dot{V}$O_2_ versus $\dot{V}$CO_2_ plot. A difference plot of $\dot{V}$CO_2_ minus $\dot{V}$O_2_ was used to identify the inflection point for the difference versus time. This method was used to cross-check the visually determined GET values [40]. The RCP was determined, through visual inspection, as the point of departure from linearity of the $\dot{V}$CO_2_ versus $\dot{V}$E relationship. The partial pressure of carbon dioxide (PCO_2_) was plotted as a function of time to determine the point of decrease in PCO_2_ to cross-check the visually inspected RCP value [5]. Two independent assessors determined the GET and RCP. Upon disagreements, a secondary criterion was used to identify a single value agreed upon by both assessors.

#### 2.3.3. Determination of the PO, HR, and RPE Associated with the GET and RCP

Linear regression was used to determine the PO, HR, and RPE associated with the GET and RCP thresholds. To determine the PO associated with the GET and RCP, PO (W) was plotted as a function of $\dot{V}$O_2_ (L·min^−1^). A linear line of best fit was used to determine the regression equation of the relationship between $\dot{V}$O_2_ and PO. The respective $\dot{V}$O_2_ (L·min^−1^) for the GET and RCP were input to the regressed equation to determine the PO associated with the GET (PO_GET_) and RCP (PO_RCP_) [r = 0.963–1.000].

To determine the HR associated with the GET and RCP, HR (b·min^−1^) was plotted as a function of $\dot{V}$O_2_ (L·min^−1^). A linear line of best fit was used to determine the regression equation of the relationship between $\dot{V}$O_2_ and HR. The respective $\dot{V}$O_2_ (L·min^−1^) for the GET and RCP were input to the regressed equation to determine the HR associated with the GET (HR_GET_) and RCP (HR_RCP_) [r = 0.962–0.998].

To determine the RPE associated with the GET and RCP, RPE was plotted as a function of $\dot{V}$O_2_ (L·min^−1^). A linear line of best fit was used to determine the regression equation of the relationship between $\dot{V}$O_2_ and RPE. The respective $\dot{V}$O_2_ (L·min^−1^) for the GET and RCP were input to the regressed equation to determine the RPE associated with the GET (RPE_GET_) and RCP (RPE_RCP_) [r = 0.944–0.998].

### 2.4. Statistical Analyses

Separate 2 (test [test 1, test 2]) × 2 (sex [male, female]) mixed-model analyses of variance (ANOVAs) were used to examine systematic error across time and potential effects of sex for the GET ( $\dot{V}$O_2_), RCP ( $\dot{V}$O_2_), PO_GET_, PO_RCP_, HR_GET_, HR_RCP_, RPE_GET_, and RPE_RCP_. Post hoc analyses for any main or interaction effects included Bonferroni-corrected paired and independent samples t-tests. Responses for the GET and RCP were examined for absolute (L·min^−1^, W), relative (mL·kg^−1^·min^−1^), and normalized (%PPO and % $\dot{V}$O_2peak_) values, while absolute (b·min^−1^ and RPE) and normalized (%HR_peak_ and %RPE_peak_) values were examined for the HR and RPE associated with the GET (HR_GET_, RPE_GET_, HR_NORM GET_, RPE_NORM GET_) and RCP (HR_RCP_, RPE_RCP_, HR_NORM RCP_, RPE_NORM RCP_).

The test–retest reliabilities for the GET, RCP, PO_GET_, PO_RCP_, HR_GET_, HR_RCP_, RPE_GET_, and RPE_RCP_ (absolute, relative, and normalized) were examined separately for each sex using an intraclass correlation coefficient (ICC_2,1_) model. The following equation was used for the ICC determination:

(1) $${\mathrm{ICC}}_{2,1}=\frac{{\mathrm{MS}}_{S}-{\mathrm{MS}}_{E}\;}{{\mathrm{MS}}_{S}+\left(k-1\right){\mathrm{MS}}_{E}+\left(\;\frac{k\left({\mathrm{MS}}_{T}-{\mathrm{MS}}_{E}\right)}{n}\right)}$$

where subjects mean square ( ${\mathrm{MS}}_{S}$) represents the variance between-subject effects, the mean square error ${(\mathrm{MS}}_{E})$ is the residual square error, representing random error arising from the trial by subjects interaction, and the tests mean square factor ( ${\mathrm{MS}}_{T})$ represents systematic variance attributed to differences between tests. The k-value is the number of tests run (k = 2) while n represents the sample size (n = 9). The ICC values were classified as follows: “excellent” (0.8–1.0), “good” (0.6–0.8), and “poor” (<0.6) [41]. The ICC value provides a measure of relative reliability [42].

The standard error of measure (SEM) was calculated as an absolute measure of reliability using the following equation:

(2) $$\mathrm{SEM}\;=\;\sqrt{{\mathrm{MS}}_{E}}$$

The minimal difference needed to be considered real (MD) was calculated to determine the testing variability that falls within 95% of the confidence interval. The following equation was used:

(3) $$\mathrm{MD}=\mathrm{SEM}\;\times \;1.96\times \sqrt{2}$$

The coefficient of variation (CV) was used to provide a normalized value of the SEM; the following equation was used:

(4) $$\mathrm{CV}=\;\frac{\mathrm{SEM}}{\mathrm{GrandMean}}\times 100$$

Based on previous recommendations [43], a CV of <10% was used as an indication of sufficient absolute reliability. However, the overall reliability of the measures was characterized by factoring the ICC value, in conjunction with the CV, SEM, and MD. Additionally, mean bias (test 1–test 2) and 95% limits of agreement (LOA) were calculated for each variable. Measures of effect size included partial eta squared (p*η*^2^) and were interpreted as 0.01 for small, 0.06 for medium, and 0.14 for large effects [44]. All analyses were conducted using IBM SPSS Statistics (v.29.0.2.0. IMB SPSS Inc., Chicago, IL, USA).

## 3. Results

### 3.1. Peak Values

The peak values (peak oxygen consumption $[\dot{V}$O_2peak_], peak power output [PPO], heart rate peak [HR_peak_], rating of perceived exertion peak [RPE_peak_]) from GXTs 1 and 2 are presented in Table 1 for descriptive purposes.

### 3.2. Gas Exchange Threshold

There were no sex x time interactions (*p* = 0.462, F = 0.567, p*η*^2^ = 0.034; *p* = 0.441, F = 0.625, p*η*^2^ = 0.017) or main effects for time (*p* = 0.247, F = 1.446, p*η*^2^ = 0.083; *p* = 0.609, F = 0.272, p*η*^2^ = 0.017) for the absolute expressions of the GET (L·min^−1^ and W, respectively). There were, however, main effects for sex (*p* = 0.005, F = 10.583, p*η*^2^ = 0.398; *p* = 0.014, F = 7.691, p*η*^2^ = 0.325) for both absolute expressions that indicated a greater absolute GET for the males (Mean ± SD: 2.37 ± 0.72 L·min^−1^; 182 ± 61 W) than the females (Mean ± SD: 1.50 ± 0.32 L·min^−1^; 118 ± 31 W). For the relative (mL·kg^−1^·min^−1^) and normalized GET (% $\dot{V}$O_2peak_, %PPO), there were no sex x time interactions (relative $\dot{V}$O_2_: *p* = 0.522, F = 0.429, p*η*^2^ = 0.026; normalized % $\dot{V}$O_2peak_: *p* = 0.089, F = 3.29, p*η*^2^ =0.170; normalized %PPO: *p* = 0.521, F = 0.430, p*η*^2^ = 0.026), main effects for time (relative $\dot{V}$O_2_: *p* = 0.297, F = 1.162, p*η*^2^ = 0.068; normalized % $\dot{V}$O_2peak_: *p* = 0.865, F = 0.03, p*η*^2^ =0.02; normalized %PPO: *p* = 0.865, F = 0.030, p*η*^2^ = 0.002), or main effects for sex (relative $\dot{V}$O_2_: *p* = 0.368, F = 0.859, p*η*^2^ =0.051, normalized % $\dot{V}$O_2peak_: *p* = 0.765, F = 0.093, p*η*^2^ =0.006; normalized %PPO: *p* = 0.521, F = 0.430, p*η*^2^ = 0.026). The Mean ± SD for test 1 and test 2, as well as the results of the reliability analyses for the GET, are presented in Table 2 and Table 3.

### 3.3. Respiratory Compensation Point

There were no sex x time interactions (*p* = 0.146, F = 2.33, p*η*^2^ = 0.127; *p* = 0.477, F = 0.529, p*η*^2^ = 0.032) or main effects for time (*p* = 0.886, F = 0.021, p*η*^2^ = 0.001; *p* = 0.756, F = 0.100, p*η*^2^ = 0.006) for the absolute expressions of the RCP (L·min^−1^ and W, respectively). There were, however, main effects for sex (*p* < 0.001, F = 23.282, p*η*^2^ = 0.593; *p* = 0.002, F = 13.545, p*η*^2^ = 0.458) for both absolute expressions that indicated a greater absolute RCP for the males (Mean ± SD: 3.10 ±0.58 L·min^−1^; 246 ± 50 W) than the females (Mean ± SD: 2.07 ±0.24 L·min^−1^; 179 ± 21 W). For the relative (mL·kg^−1^·min^−1^) and normalized RCP (% $\dot{V}$O_2peak_, %PPO), there were no sex x time interactions (relative $\dot{V}$O_2_: *p* = 0.150, F = 2.288, p*η*^2^ = 0.125; normalized % $\dot{V}$O_2peak_: *p* = 0.783, F = 0.079, p*η*^2^ = 0.005; normalized %PPO: *p* = 0.456, F = 0.584, p*η*^2^ = 0.035) or main effects for time (relative $\dot{V}$O_2_: *p* = 0.761, F = 0.096, p*η*^2^ = 0.006; normalized % $\dot{V}$O_2peak_: *p* = 0.103, F = 2.984, p*η*^2^ = 0.157; normalized %PPO: *p* = 0.132, F = 2.520, p*η*^2^ = 0.136). There was, however, a main effect for sex for the RCP normalized to $\dot{V}$O_2peak_ (*p* < 0.001, F = 18.351, p*η*^2^ = 0.534), but not for the RCP normalized to PPO (*p* = 0.116, F = 2.757, p*η*^2^ = 0.147) or for the relative values (relative $\dot{V}$O_2_: *p* = 0.419, F = 0.688, p*η*^2^ = 0.41). The normalized (% $\dot{V}$O_2peak_) RCP was significantly greater for the females (93 ± 4 % $\dot{V}$O_2peak_) compared to the males (87 ± 3 % $\dot{V}$O_2peak_). The Mean ± SD for test 1 and test 2, as well as the results of the reliability analyses for the RCP, are presented in Table 2 and Table 4.

### 3.4. Heart Rate (HR) at the GET and RCP

There was no sex x time interaction (*p* = 0.882, F = 0.052, p*η*^2^ = 0.003), main effect for time (*p* = 0.535, F = 0.401, p*η*^2^ = 0.024), or main effect for sex (*p* = 0.264, F = 1.338, p*η*^2^ = 0.077) for the HR_GET_ (b·min^−1^). There was no sex x time interaction (*p* = 0.474, F = 0.538, p*η*^2^ = 0.033) or main effect for time (*p* = 0.862, F = 0.031, p*η*^2^ = 0.002) at the HR_RCP_ (b·min^−1^). However, there was a main effect for sex (*p* = 0.040, F = 5.02, p*η*^2^ = 0.239) that indicated a greater HR_RCP_ for the females (Mean ± SD: 175 ± 10 b·min^−1^) than the males (Mean ± SD: 167 ± 9 b·min^−1^). The HR associated with the GET and RCP normalized (NORM) to the peak HR (%HR_peak_) displayed no sex x time interactions (HR_NORM GET_: *p* = 0.678, F = 0.179, p*η*^2^ = 0.011; HR_NORM RCP_: *p* = 0.944, F = 0.005, p*η*^2^ = 0.000) or main effect for time (HR_NORM GET_: *p* = 0.835, F = 0.045, p*η*^2^ = 0.003: HR_NORM RCP_: *p* = 0.297, F = 1.160, p*η*^2^ = 0.068). There was, however, a main effect for sex for the HR_NORM RCP_ (*p* < 0.001, F = 17.255, p*η*^2^ = 0.519), but not for the HR_NORM GET_ (*p* = 0.278, F = 1.260, p*η*^2^ = 0.073). The HR_NORM RCP_ was significantly greater for the females (97 ± 2% HR_peak_) compared to the males (93 ± 3% HR_peak_). The Mean ± SD for test 1 and test 2, as well as the results of the reliability analyses for the HR at the GET and RCP, are presented in Table 5.

### 3.5. Rating of Perceived Exertion (RPE) at the GET and RCP

There was no sex x time interaction (*p* = 0.536, F = 0.400, pη^2^ = 0.024), main effect for time (*p* = 0.076, F = 3.600, pη^2^ = 0.184), or main effect for sex (*p* = 0.837, F = 0.044, pη^2^ = 0.003) at the RPE_GET_. There was no sex × time interaction (*p* = 0.845, F = 0.040, pη^2^ = 0.002), main effect for time (*p* = 0.092, F = 3.208, pη^2^ = 0.167), or main effect for sex (*p* = 0.079, F = 3.531, pη^2^ = 0.181) for the RPE_RCP_. The RPE associated with the GET and RCP normalized to the peak RPE showed no sex x time interactions (GET: *p* = 0.361, F = 0.886, pη^2^ = 0.052; RCP: *p* = 0.584, F = 0.376, pη^2^ = 0.023) or main effect for time (GET: *p* = 0.444, F = 0.615, pη^2^ = 0.037; RCP: *p* = 0.134, F = 2.488, pη^2^ = 0.135). However, there was a main effect for sex at the normalized RPE associated with the RCP (*p* = 0.001, F = 15.836, pη^2^ = 0.497), but not the GET (*p* = 0.805, F = 0.063, pη^2^ = 0.004). The normalized RPE associated with the RCP was significantly greater for the females (92 ± 4 %RPE_peak_) than for the males (86 ± 4% RPE_peak_). The Mean ± SD for test 1 and test 2, as well as the results of the reliability analyses for the RPE at the GET and RCP, are presented in Table 6.

## 4. Discussion

### 4.1. Reliability of Fatigue Thresholds

The findings from this study highlight that PO and $\dot{V}$O_2_ serve as robust methods of identifying the GET and RCP because of their high absolute and relative reliabilities across absolute and relative expressions for males and females. The reliability of fatigue thresholds and their associated perceptual and physiological anchors is important for ensuring effective exercise prescription techniques. This study investigated the reliability of two fatigue thresholds (GET and RCP) and their associated HR and RPE values from a test–retest GXT protocol in males and females. The primary findings of this study included the following: (1) absolute (W, L·min^−1^) and relative (mL·kg^−1^·min^−1^) expression of the GET and RCP demonstrated “excellent” reliability across males and females, but normalized responses tended to be less reliable; (2) HR at the GET expressed in absolute (b·min^−1^) terms and normalized to peak (% of HR_peak_) demonstrated “excellent” to “good” reliability for males and females, respectively, while demonstrating predominately “poor” reliability at the RCP for both sexes; (3) associated RPE values expressed in absolute (Borg’s 6-20) terms and normalized to peak (% of RPE_peak_) lacked consistency across sexes and fatigue thresholds; and (4) males demonstrated greater absolute PO and $\dot{V}$O_2_ values at the GET and RCP, while females demonstrated a greater normalized $\dot{V}$O_2_ response at the RCP as well as absolute and normalized HR and RPE responses at the RCP. Overall, expression of the fatigue thresholds via PO (W) and $\dot{V}$O_2_ (L·min^−1^, mL·kg^−1^·min^−1^) tended to demonstrate greater reliability than perceptual and physiological metrics of RPE and HR, respectively.

In the current study, the GET demonstrated “excellent” reliability across absolute, relative, and normalized PO (W) and $\dot{V}$O_2_ (L·min^−1^, mL·kg^−1^·min^−1^) for males (0.913–0.978), whereas females demonstrated “good” to “excellent” reliability (0.753–0.938). These findings were consistent with those of O’Malley et al. [17]**,** who demonstrated “excellent” reliability for the GET expressed as relative $\dot{V}$O_2_ (R = 0.929; ml·kg^−1^·min^−1^) and absolute PO (R = 0.957; W) in a heterogeneous sample of seven males and one female. The results of van der Zwaard et al. [19] similarly demonstrated “excellent” reliability (R = 0.97) of the VT_1_ expressed in absolute terms of $\dot{V}$O_2_ (L·min^−1^). It is important to note that the GET and VT_1_ are often used synonymously in the literature; however, they are derived from separate parameters and are not the same threshold. VT_1_ was first proposed by Wasserman et al. [45] and relies on ventilatory equivalents ( $\dot{V}$Es) rather than expired gases ( $\dot{V}$O_2_, $\dot{V}$CO_2_) used for GET determination. The authors [19], however, determined VT_1_ using the same V-slope method [5] that was used in this study to derive the GET. In addition, previous studies [46,47] have reported no significant differences in mean values between the GET and VT_1_ and suggested they reflect a similar point of transition from the moderate to the heavy exercise intensity domains. The normalized (% of PPO) PO at the GET demonstrated unique reliability differences between males and females in this study. Specifically, the sample of male subjects reflected “excellent” reliability (R = 0.913), while the sample of females demonstrated “good” reliability (R = 0.753). The ICC provides a measure of relative reliability [42] and should be used in conjunction with the SEM, MD, and CV to assess reliability. The CV and SEM reflect measures of absolute reliability [42], where a CV <10% may suggest acceptable agreement between tests [43]. The SEM of females was similar to that of males (M: 4%, F: 6%), and the CV was at or below 10.0%. Taken together, these metrics suggested that the GET in this study demonstrated strong reliability across absolute, relative, and normalized expressions of PO and $\dot{V}$O_2_, consistent with previous evidence [17,19].

The “excellent” relative reliability for the absolute and relative expressions of PO and $\dot{V}$O_2_ at the RCP for males (R = 0.951–0.975) and females (R = 0.816–0.945) in this study was consistent with the “excellent” reliability reported previously for absolute and relative expressions of the VT_2_ for cycle ergometry [15,18] and treadmill running [16]. Similar to the GET and VT_1_, the RCP and VT_2_ are used synonymously and have been suggested to be the transition point for the heavy to severe intensity domains [7]. An important consideration in this study was the assessment of reliability of each threshold expressed as absolute, relative, and normalized $\dot{V}$O_2_ for cycle ergometry. Although the absolute and relative expressions of the RCP demonstrated “excellent” reliability in this study, the reliability was “poor” when normalized to $\dot{V}$O_2peak_. Similarly, Prud’Homme et al. [14] demonstrated “excellent” reliability for the $\dot{V}$O_2_ at VT_2_ when expressed in absolute (R = 0.90; L·min^−1^) and relative (R = 0.93; mL·kg·min^−1^) terms, but “poor” reliability (R = 0.27; % $\dot{V}$O_2peak_) was reported for the normalized values. Prud’Homme et al. [14] suggested that the decreased reliability for normalized values may be attributed to the forced restriction of variation when variables are bound to a 0–100 scale. Normalization may also influence the interpretation of the CV. Absolute and relative expressions of variables generally display normative variability, which leads to generally high reliability and a CV between 5 and 10% [14,48]. However, unlike absolute and relative expression, normalization constrains the variability in data (e.g., 0–100%). Therefore, a low ICC and CV may reflect this forced constraint that reduces between and within-subjects variability. Based on the current and previous findings, absolute and relative expression of thresholds may serve as a more reliable and sensitive method for tracking changes in cardiorespiratory fitness amongst individuals compared to normalized responses.

A unique aspect of this study was the simultaneous examination of reliability of the HR and RPE associated with the GET and RCP. Although these thresholds are most commonly expressed as $\dot{V}$O_2_ and PO, measures of HR and RPE may be more accessible for application to exercise prescription as they do not require the use of a metabolic cart or power meter in the field, serving as a cost-effective method in measuring exercise intensity. The absolute and normalized responses of HR and RPE at the GET reflected “excellent” relative reliability for males (R = 0.855–0.886), but “good” to “poor” relative reliability for HR (R = 0.670–0.679) and RPE (R = 0.368–0.573), respectively, for female subjects. The findings for the HR associated with the GET were generally consistent with those of O’Malley et al. [17] and Sempere-Ruiz et al. [33]**,** who demonstrated that the HR associated with the GET or VT_1_ demonstrated “good” relative reliability (R = 0.668–0.79; b·min^−1^). No previous studies have reported the reliability of the RPE associated with the GET using a test–retest GXT methodology. The “poor” relative reliability demonstrated by female subjects for the absolute and normalized RPE at the GET was associated with slightly greater CVs (6.6–7.7%) compared to males (4.4–5.5%), but both were below the 10% threshold suggested to demonstrate absolute reliability [43]. The low ICC accompanied by CVs <10% may be explained by further data constraints due to normalization, as discussed earlier regarding the normalized $\dot{V}$O_2_ at the RCP. Taken together, the current findings suggested that the HR associated with the GET demonstrated acceptable reliability for males and females, while the RPE at the GET demonstrated acceptable absolute reliability for both sexes, but limited relative reliability for females.

Contrary to the GET, the RCP demonstrated predominantly “poor” relative reliability across the associated HR and RPE expressions for males and females, but acceptable absolute reliability (CV less than or equal to ~5%) with no systematic error. Previously, “excellent” relative reliability has been reported for the HR associated with VT_2_ or the anerobic threshold (R = 0.91–0.92; b·min^−1^) [18,34], but no previous studies have reported relative or absolute reliability of the RPE associated with the RCP. The lower relative reliability for the HR associated with the RCP in this study, compared to previously reported values, may be related to the subject’s position rank from test 1 to test 2. Weir [42] described that relative reliability (ICC) is sensitive to the consistency of the individual position rank order from test to test. As displayed in Table 5, eight of the nine male subjects’ respective positioning changed. Similarly, eight of the nine female subjects’ respective positioning changed from test 1 to test 2. Therefore, even though there was low error (CV: 4.1–5.3%), suggesting strong absolute reliability, the relative reliability may have been affected by greater position change, change in individual subject rank order between tests, or the relatively small sample size (M-n = 9, F-n = 9). The “poor” reliability demonstrated in this study for the HR and RPE at the RCP may also be related to compounding error in identifying associated values from regression equations to predict HR and RPE. Additionally, day-to-day variability in cardiovascular [49] and perceptual responses [50] may affect the subjects’ position rank order from test to test. Poor sleep [49], psychological stressors [51], and the menstrual cycle [50] may impact cardiovascular responses, whereas day-to-day fluctuations in perceptual responses may be affected by a combination of the menstrual cycle [50], locomotor effort [52], respiratory effort [53], and central motor command [54]. The test–retest trials were performed within 2–5 days, which likely limited changes in menstrual cycle phase and the influence of hormonal shifts on responses in this study. It is also possible that the lower reliability for the RCP relative to the GET reflects greater physiological perturbations, such as H^+^ accumulation [55], hyperventilation [55], and increased contribution of groups III and IV muscle afferents [56] that may inform cardiovascular and perceptual responses at greater intensities (i.e., RCP) that are more limited at the GET. In addition, the limitation presented previously regarding normalized variable expression may also be present when analyzing values associated with the RPE. Because RPE values range from 6 to 20, there may be external constraints on this variable, prior to normalization that is not present with PO, $\dot{V}$O_2_, or HR. Additionally, with the RCP occurring near an individual’s point of volitional fatigue, the range of RPE values selected may be smaller than that of a lower-intensity threshold, such as the GET, constraining the between-subject variability and reducing the ICC. Future research should investigate larger continuous-intensity measurement scales, such as the Borg CR100 [57]**,** to reduce the variable constraint at greater intensities. The associated HR and RPE may be more accessible methods for prescribing exercise at the GET and RCP, although it should be recognized that the lower relative reliability may make them somewhat less sensitive for targeting the desired physiological responses that occur at the GET and RCP to improve cardiorespiratory fitness.

### 4.2. Sex Differences in Fatigue Thresholds

The primary aim of this study was to investigate the reliability of perceptual and cardiovascular variables associated with the GET and RCP in males and females. Through this analysis, significant mean differences in absolute and normalized responses were identified for males and females. The greater absolute PO and $\dot{V}$O_2_ at the GET and RCP for males compared to females in this study was consistent with the literature [15,18] and has been attributed to the greater overall body mass and muscle mass for males compared to females [24,25]. Sex differences in relative (mL·kg·min^−1^) and normalized (% $\dot{V}$O_2peak_) responses may persist due to factors such as greater hemoglobin concentration [23], testosterone [58], or proportion of skeletal muscle mass within fat-free body mass [59] for males compared to females. In this study, the relative $\dot{V}$O_2_ at the GET and RCP reflected slightly higher, but not significantly different, mean values for males compared to females. The findings of Rodríguez-Barbero et al. [22] demonstrated significantly greater $\dot{V}$O_2_ responses in males than females at VT_1_ (M: 51.15 ± 4.67, F: 44.23 ± 5.64 mL·kg^−1^·min^−1^) and VT_2_ (M: 62.83 ± 5.80, F: 53.60 ± 6.17 mL·kg^−1^·min^−1^). These findings, however, were demonstrated in treadmill running, not during cycling. Interestingly, there were no significant differences between males and females for normalized $\dot{V}$O_2_ (% of $\dot{V}$O_2peak_) at the GET, while the normalized $\dot{V}$O_2_ at the RCP reflected greater values for females (93 ± 4%) than males (87 ± 3%) in this study. It is possible these differences reflect the nature of the 30 W incremental protocol, whereby females experienced greater relative increases in PO and may have been limited by peripheral factors (e.g., muscular strength) more than central (e.g., cardiac output) at the end of the test. It is also possible that the greater normalized RCP response for females reflected morphological differences. A greater type I fiber type cross-sectional area has been demonstrated in females compared to males [60], which was accompanied by greater fat oxidation during 90 min cycling rides at 60% of $\dot{V}$O_2peak_ [60]. These morphological differences may have reflected greater aerobic contribution and limited anaerobic glycolytic activity, which delayed the RCP for females compared to males. Future studies should further explore possible sex differences in normalized threshold responses under various graded exercise test protocols (i.e., ramp or <30 W stages) and examine morphological characteristics.

The greater absolute HR at both thresholds and the greater normalized HR and RPE values at the RCP for females compared to males highlight interesting sex differences in cardiovascular and perceptual responses. The greater HR response for females compared to males is likely related to relative differences in stroke volume [61]. Previous evidence suggests females may have as much as a 23% lower stroke volume than males [27]. Therefore, to compensate for the lower stroke volume, a greater HR response is required to meet the cardiac output demands at the GET and RCP for females compared to males. Rodríguez-Barbero et al. [22] demonstrated significantly greater absolute and normalized HR responses in females than males at VT_1_ (HR_absolute_: 160 ± 10, 167 ± 14 b·min^−1^; HR_NORM_: 86 ± 4, 88 ± 3%) but not at VT_2_ during treadmill running. It is possible that the greater normalized HR and RPE at the RCP for females in this study is attributed to a delayed occurrence of the RCP from greater aerobic energy contributions [60] and greater oxidative mitochondrial efficiency [62]. The delayed attainment of reaching the RCP, however, prolongs the duration of time spent within the heavy-intensity domain, which has been shown to elicit greater metabolic byproduct accumulations that contribute to peripheral and central fatigue [63]. Therefore, it is possible that a greater accumulation of fatigue may underpin the higher HR and RPE responses at the RCP for females, while the delayed attainment of reaching the RCP may explain the significantly greater normalized RPE and HR responses at the RCP. Future studies should further explore these potential mechanisms underlying sex differences in cardiovascular and perceptual responses. These findings highlight that the way a physiological variable is expressed may have important nuances in prescription to males and females.

## 5. Limitations

This study should not be considered without its limitations. Regression equations were used to determine the associated values of PO [r^2^ = 0.928–1.000], HR [r^2^ = 0.926–0.998], and RPE [r^2^ = 0.892–0.998] and may contain error as their variance (r^2^) is not entirely explained by $\dot{V}$O_2_. In addition, the thresholds were independently verified by two investigators; however, the investigators were not blinded to session order or sex, which may introduce unintentional bias. Although individuals underwent a familiarization test and standardized visit times (±2 h), day-to-day fluctuations in responses may be explained by factors that were not controlled in this study, including menstrual cycle [50], sleep [49], psychological stressors [51], and cardiopulmonary/muscular effort [52,53]. Additionally, factors that were not standardized across visits that may impact cardiovascular and perceptual responses include hydration, glycogen storage, menstrual cycle, and sleep. The relatively small sample size (n = 9 males and n = 9 females) may limit the generalizability of sex differences identified in this study. Future studies should further examine potential sex differences in thresholds across larger samples.

## 6. Conclusions

The findings of this study highlight that PO and $\dot{V}$O_2_ serve as the optimal criteria for prescribing intensity due to their strong absolute and relative reliabilities across males and females at the GET and RCP. HR and RPE, however, may serve as more accessible options than PO and $\dot{V}$O_2_ in prescribing exercise. Although the ICCs were poor in some cases, likely due to the degree of variability within the sample, these variables demonstrated acceptable absolute reliability, as reflected by low SEMs and CVs less than or equal to 10% with no systematic error (no mean differences test–retest). Therefore, HR and RPE have applicability to monitor and prescribe exercise intensities at the GET and RCP. Due to their relative reliability, the sensitivity at which they can target the desired metabolic responses at the GET and RCP should be considered. Additionally, absolute (W, L·min^−1^, b·min^−1^, Borg 6-20) and relative (mL·kg·min^−1^) expression of the GET and RCP systematically outperformed the relative reliability of normalized (%_peak_) expressions. When prescribing individualized intensities, absolute or relative expression of variables should be used due to their strong absolute and relative reliability. Normalized expressions of variables are useful for comparing responses across sexes, domains, and fitness levels. However, caution should be used when prescribing exercise from the grand mean of normalized expressions due to the variability in where a fatigue threshold may fall in relation to the individual’s maximum. Lastly, this study demonstrated significant differences in responses at the GET and RCP between males and females. These findings suggested a need for future studies to explore the sex-dependent responses in cardiovascular and perceptual parameters, particularly at intensities greater than the GET.

## Figures and Tables

**Table 1:** Mean ± SD peak responses from the graded exercise tests.

	Males		Females	
	T1	T2	T1	T2
$\dot{V}$ **O_2peak_ (L·min^−1^)**	3.56 ± 0.60	3.55 ± 0.71	2.27 ± 0.25	2.18 ± 0.26
$\dot{V}$ **O_2peak_ (mL·kg^−1^·min^−1^)**	44.77 ± 7.69	44.69 ± 8.89	39.59 ± 7.66	38.15 ± 8.33
**PPO (W)**	287 ± 57	283 ± 56	203 ± 23	202 ± 22
**HR_peak_ (b·min^−1^)**	178 ± 8	179 ± 4	182 ± 6	179 ± 10
**RPE_peak_ (Borg’s 6-20)**	19 ± 1	19 ± 1	19 ± 1	19 ± 1

Test 1 (T1), test 2 (T2), peak oxygen consumption ( $\dot{V}$O_2peak_), peak power output (PPO), heart rate peak (HR_peak_), rating of perceived exertion peak (RPE_peak_).

**Table 2:** Individual and Mean ± SD responses for power output (PO) at the gas exchange threshold (GET) and respiratory compensation point (RCP) for the males (M) and females (F) from test 1 (T1) and test 2 (T2), as well as the results of the reliability analyses.

Subject	T1 (PO_GET_)	T2 (PO_GET_)	T1 (GET _%PPO_)	T2 (GET _%PPO_)	T1 (PO_RCP_)	T2 (PO_RCP_)	T1 (RCP _%PPO_)	T2 (RCP _%PPO_)
M1	174	142 ^+^	67	55 ^+^	224	229	86	88
M2	100	105	50	53	173	173	87	86
M3	97	89	42	39	193	178	84	78
M4	165	170	63	65	203	223	78	86
M5	297	297	78	78	329	332	87	87
M6	228	227	71	71	266	284	83	89
M7	201	186	58	58	299	291	85	91
M8	204	216	70	67	241	271	83	85
M9	194	189	67	73	256	236	88	91
**Mean ± SD**	**185 ± 62**	**180 ± 64**	**63 ± 11**	**62 ± 12**	**243 ± 51**	**246 ± 53**	**85 ± 3**	**86 ± 4**
**ICC**	**0.978**		**0.913**		**0.951**		**0.236**	
**[95% CI]**	**[0.915–0.995]**		**[0.669–0.980]**		**[0.809–0.989]**		**[−0.335–0.736]**	
**SEM**	**9 W**		**4%**		**12 W**		**3%**	
**MD**	**26 W**		**10%**		**33 W**		**8%**	
**CV**	**5.0%**		**6.1%**		**4.8%**		**3.5%**	
**Mean ± SD**	182 ± 61 W *		**63 ± 11%**		246 ± 50 W *		**86 ± 4%**	
**Bias (SD) **	**4.53 (12.21)**		**0.94 (4.78)**		**−3.63 (15.64)**		**−2.12 (3.86)**	
**[LOA]**	**[−19.40–28.45]**		**[−8.43–10.32]**		**[−34.29–27.02]**		**[−9.68–5.45]**	
F1	133	142	67	71	179	191	89	95
F2	90	73	53	43	139	144	82	85
F3	134	147	58	64	196	199	85	87
F4	80	86	40	43	171	167	86	84
F5	125	98	62	49	178	168	89	84
F6	78	82	46	48	163	159	96	93
F7	169	165	73	72	201	205	87	89
F8	109	130	54	65	167	178	84	89
F9	137	140	60	70	215	185 ^+^	93	92
**Mean ± SD**	**117 ± 30**	**118 ± 34**	**63 ± 10**	**57 ± 12**	**179 ± 23**	**177 ± 20**	**88 ± 4**	**89 ± 4**
**ICC**	**0.902**		**0.753**		**0.832**		**0.628**	
**[95% CI]**	**[0.623–0.977]**		**[0.226–0.939]**		**[0.414–0.960]**		**[−0.014–0.902]**	
**SEM**	**11 W**		**6%**		**9 W**		**3%**	
**MD**	**29 W**		**16%**		**25 W**		**7%**	
**CV**	**8.9%**		**10.0%**		**5.1%**		**3.0%**	
**Mean ± SD**	**118 ± 31 W**		**58 ± 11%**		**178 ± 21 W**		**88 ± 4%**	
**Bias (SD) **	**−1.00 (13.99)**		**−1.30 (7.79)**		**1.50 (12.07)**		**−0.76 (3.44)**	
**[LOA]**	**[−28.42–26.42]**		**[−16.56–13.96]**		**[−22.16–25.16]**		**[−7.50–5.98]**	

Note: ICC = intraclass correlation coefficient, CI = confidence interval, SEM = standard error of the measurement, MD = minimal difference to be considered real, CV = coefficient of variation, Bias = Test 1–Test 2, SD = standard deviation, LOA = limits of agreement, %PPO = percent of peak power output: PO measured in W. * Indicates a significantly greater absolute PO at the GET and RCP for the males compared to females. ^+^ Indicates the difference between test 1 and test 2 exceeded the MD.

**Table 3:** Individual and Mean ± SD responses for the $\;\dot{V}$O_2_ at the gas exchange threshold (GET) for the males (M) and females (F) from test 1 (T1) and test 2 (T2), as well as the results of the reliability analyses.

Subject	T1 GET (L·min^−1^)	T2 GET (L·min^−1^)	T1 GET (mL·kg^−1^·min^−1^)	T2 GET (mL·kg^−1^·min^−1^)	T1 GET ($\%\dot{V}$O_2peak_)	T2 GET ($\%\dot{V}$O_2peak_)
M1	2.20	1.81 ^+^	29.04	23.92 ^+^	68	62
M2	1.44	1.42	20.71	20.48	55	59
M3	1.53	1.45	15.30	14.45	47	47
M4	2.08	2.20	27.02	28.67	67	66
M5	3.77	3.81	43.82	44.33	82	79
M6	2.99	2.91	41.03	39.89	72	70
M7	2.49	2.31	29.34	27.17	65	61
M8	2.67	2.65	33.65	33.37	75	72
M9	2.44	2.48	32.70	33.17	67	67
**Mean ± SD**	**2.40 ± 0.72**	**2.34 ± 0.75**	**30.29 ± 8.98**	**29.49 ± 9.36**	**66 ± 10**	**65 ± 9**
**ICC**	**0.978**		**0.976**		**0.941**	
**[95% CI]**	**[0.911–0.995]**		**[0.905–0.994]**		**[0.746–0.986]**	
**SEM**	**0.10 L·min^−1^**		**1.38 mL·kg^−1^·min^−1^**		**2%**	
**MD**	**0.29 L·min^−1^**		**3.83 mL·kg^−1^·min^−1^**		**6%**	
**CV**	**4.4%**		**4.6%**		**3.3%**	
**Mean ± SD**	2.37 ± 0.72 L·min^−1^ *		**29.89 ± 9.91 mL·kg^−1^·min^−1^**		**65 ± 9%**	
**Bias (SD)**	**0.06 (0.14)**		**0.80 (1.84)**		**1.78 (2.86)**	
**[LOA]**	**[−0.22–0.34]**		**[−2.82–4.41]**		**[−3.81–7.38]**	
F1	1.67	1.74	31.26	32.55	69	76
F2	1.21	1.10	18.54	16.91	60	55
F3	1.76	1.77	28.95	29.16	71	74
F4	1.10	1.13	21.94	22.41	54	56
F5	1.69	1.44	25.16	21.34 ^+^	72	65
F6	1.03	1.07	19.21	20.06	54	59
F7	2.06	1.99	43.88	42.54	78	74
F8	1.43	1.62	22.31	25.18	66	75
F9	1.60	1.56	25.42	24.77	67	76
**Mean ± SD**	**1.51 ± 0.34**	**1.49 ± 0.33**	**26.30 ± 7.81**	**26.10 ± 7.75**	**66 ± 8**	**68 ± 9**
**ICC**	**0.938**		**0.972**		**0.769**	
**[95% CI]**	**[0.753–0.986]**		**[0.883–0.994]**		**[0.307–0.942]**	
**SEM**	**0.10 L·min^−1^**		**1.37 mL·kg^−1^·min^−1^**		**4%**	
**MD**	**0.25 L·min^−1^**		**3.80 mL·kg^−1^·min^−1^**		**11%**	
**CV**	**6.0%**		**5.2%**		**6.1%**	
**Mean ± SD**	**1.50 ± 0.32 L·min^−1^**		**26.20 ± 7.55 mL·kg^−1^·min^−1^**		**67 ± 8%**	
**Bias (SD)**	**−0.07 (0.12)**		**0.19 (1.83)**		**−2.16 (5.45)**	
**[LOA]**	**[−0.21–0.24]**		**[−3.39–3.77]**		**[−12.83–8.51]**	

Note: ICC = intraclass correlation coefficient, CI = confidence interval, SEM = standard error of the measurement, MD = minimal difference to be considered real, CV = coefficient of variation, Bias = Test 1–Test 2, SD = standard deviation, LOA = limits of agreement, % $\dot{V}$ O_2peak_ = percent of maximal oxygen consumption rate. * Indicates a significantly greater absolute GET for the males compared to the females. ^+^ Indicates the difference between test 1 and test 2 exceeded the MD.

**Table 4:** Individual and Mean ± SD responses for the respiratory compensation point (RCP) for the males (M) and females (F) from test 1 (T1) and test 2 (T2), as well as the results of the reliability analyses.

Subject	T1 RCP (L·min^−1^)	T2 RCP (L·min^−1^)	T1 RCP (mL·kg^−1^·min^−1^)	T2 RCP (mL·kg^−1^·min^−1^)	T1 RCP $(\%\dot{V}$O_2peak_)	T2 RCP $(\%\dot{V}$O_2peak_)
M1	2.78	2.77	36.66	36.63	86	94 ^+^
M2	2.28	2.17	32.85	31.26	87	90
M3	2.69	2.61	26.89	26.06	83	84
M4	2.52	2.83 ^+^	32.72	36.78 ^+^	81	85
M5	4.16	4.25	48.31	49.43	90	88
M6	3.50	3.64	48.01	50.01	84	88
M7	3.44	3.49	40.49	41.08	90	92
M8	3.10	3.20	39.16	40.31	87	87
M9	3.23	3.14	43.20	42.08	89	85
**Mean ± SD**	**3.08 ± 0.58**	**3.12 ± 0.62**	**38.70 ± 7.21**	**39.29 ± 7.77**	**86 ± 3**	**88 ± 3**
**ICC**	**0.975**		**0.972**		**0.375**	
**[95% CI]**	**[0.900–0.994]**		**[0.892–0.994]**		**[−0.229–0.804]**	
**SEM**	**0.09 L·min^−1^**		**1.24 mL·kg^−1^·min^−1^**		**3%**	
**MD**	**0.26 L·min^−1^**		**3.44 mL·kg^−1^·min^−1^**		**7%**	
**CV**	**3.1%**		**3.2%**		**2.9%**	
**Mean ± SD**	3.10 ± 0.58 L·min^−1^ *		**39.00 ± 7.28 mL·kg^−1^·min^−1^**		**87 ± 3%**	
**Bias (SD)**	**−0.05 (0.13)**		**−0.59 (1.66)**		**−1.73 (3.47)**	
**[LOA]**	**[−0.30–0.20]**		**[−3.84–2.65]**		**[−8.52–5.06]**	
F1	2.16	2.23	40.37	41.67	89	97 ^+^
F2	1.69	1.76	25.93	26.95	84	88
F3	2.33	2.25	38.47	37.15	95	94
F4	1.91	1.87	37.86	37.16	93	93
F5	2.23	2.09	33.15	31.07	95	95
F6	1.82	1.71	33.99	32.05	96	94
F7	2.41	2.43	51.48	51.88	91	90
F8	1.99	2.08	30.99	32.34	92	96
F9	2.33	1.95 ^+^	37.01	30.86 ^+^	98	95
**Mean ± SD**	**2.10 ± 0.26**	**2.04 ± 0.24**	**36.58 ± 7.11**	**35.68 ± 7.48**	**92 ± 4**	**94 ± 3**
**ICC**	**0.816**		**0.945**		**0.519**	
**[95% CI]**	**[0.416–0.954]**		**[0.792–0.987]**		**[−0.157–0.866]**	
**SEM**	**0.11 L·min^−1^**		**1.69 mL·kg^−1^·min^−1^**		**3%**	
**MD**	**0.29 L·min^−1^**		**4.68 mL·kg^−1^·min^−1^**		**7%**	
**CV**	**5.1%**		**4.7%**		**2.7%**	
**Mean ± SD**	**2.07 ± 0.24 L·min^−1^**		**36.13 ± 7.10 mL·kg^−1^·min^−1^**		**93 ± 4% ^#^**	
**Bias (SD)**	**0.06 (0.14)**		**0.90 (2.25)**		**−1.25 (3.42)**	
**[LOA]**	**[−0.22–0.33]**		**[−3.51–5.31]**		**[−7.96–5.47]**	

Note: ICC = intraclass correlation coefficient, CI = confidence interval, SEM = standard error of the measurement, MD = minimal difference to be considered real, CV = coefficient of variation, CE = constant error, LOA = limits of agreement. * Indicates a significantly greater absolute RCP for the males compared to the females. ^#^ Indicates a significantly greater normalized RCP (% of $\dot{V}$O_2peak_) for the females compared to the males. ^+^ Indicates the difference between test 1 and test 2 exceeded the MD.

**Table 5:** Individual and Mean ± SD responses of heart rate (HR) for the males (M) and females (F) from test 1 (T1) and test 2 (T2), as well as the results of the reliability analyses.

Subject	T1 (HR_GET_)	T2 (HR_GET_)	T1 (GET _%HR peak_)	T2 (GET _%HR peak_)	T1 (HR_RCP_)	T2 (HR_RCP_)	T1 (RCP _%HRpeak_)	T2 (RCP _%HR peak_)
M1	127	134	74	74	152	171	89	95
M2	147	135	79	74	180	173	97	94
M3	121	117	64	65	171	160	91	89
M4	145	141	86	79	163	165	96	92
M5	168	166	90	90	178	175	95	95
M6	139	134	80	79	155	157	89	92
M7	143	137	79	76	170	171	95	95
M8	139	151	82	85	152	170	89	96
M9	144	150	78	83	172	170	94	95
**Mean ± SD**	**141 ± 13**	**141 ± 13**	**79 ± 7**	**79 ± 7**	**166 ± 11**	**168 ± 6**	**93 ± 3**	**94 ± 2**
**ICC**	**0.855**		**0.866**		**0.321**		**0.073**	
**[95% CI]**	**[0.481–0.965]**		**[0.523–0.968]**		**[−0.440–0.979]**		**[−0.643–0.684]**	
**SEM**	**5 b·min^−1^**		**3%**		**7 b·min^−1^**		**3%**	
**MD**	**15 b·min^−1^**		**8%**		**20 b·min^−1^**		**7%**	
**CV**	**3.8%**		**3.6%**		**4.4%**		**2.9%**	
**Mean ± SD**	**141 ± 13 b·min^−1^**		**79 ± 7%**		**167 ± 9 b·min^−1^**		**93 ± 2%**	
**Bias (SD)**	**0.85 (7.06)**		**0.74 (3.90)**		**−2.02 (9.84)**		**−0.94 (3.53)**	
**[LOA]**	**[−12.99–14.70]**		**[−6.91–8.38]**		**[−21.29–17.26]**		**[−7.68–5.98]**	
F1	156	162	84	87	178	185	96	100
F2	134	121	78	73	156	157	91	94
F3	148	167	79	87	176	188	94	99
F4	134	134	75	74	174	173	97	96
F5	156	139	86	79	177	169	98	96
F6	145	137	79	76	182	180	99	100
F7	169	166	91	90	183	182	99	98
F8	157	160	84	87	182	180	98	98
F9	143	139	83	88	175	158^+^	101	99
**Mean ± SD**	**149 ± 11**	**148 ± 16**	**82 ± 5**	**82 ± 7**	**176 ± 8**	**175 ± 12**	**97 ± 3**	**98 ± 2**
**ICC**	**0.737**		**0.679**		**0.670**		**0.480**	
**[95% CI]**	**[0.192–0.934]**		**[0.043–0.919]**		**[0.046–0.916]**		**[−0.203–0.852]**	
**SEM**	**8 b·min^−1^**		**3%**		**6 b·min^−1^**		**2%**	
**MD**	**21 b·min^−1^**		**10%**		**16 b·min^−1^**		**5%**	
**CV**	**5.1%**		**4.2%**		**3.3%**		**1.9%**	
**Mean ± SD**	148 ± 14 b·min^−1^ *		**82 ± 6%**		175 ± 10 b·min^−1^ *		**97 ± 2% ^#^**	
**Bias (SD)**	**1.78 (9.86)**		**−0.28 (4.68)**		1.36 (8.04)		**−0.78 (2.26)**	
**[LOA]**	**[−17.56–21.11]**		**[−9.45–8.88]**		[−14.39–17.11]		**[−5.21–3.64]**	

Note: ICC = intraclass correlation coefficient, CI = confidence interval, SEM = standard error of the measurement, MD = minimal difference to be considered real, CV = coefficient of variation, Bias = Test 1–Test 2, SD = standard deviation, LOA = limits of agreement. * Indicates a significantly greater absolute HR at the RCP for the females compared to the males. ^#^ Indicates a significantly greater normalized RCP (% of HR_peak_) for the females compared to the males.

**Table 6:** Individual and Mean ± SD responses of rating of perceived exertion (RPE) for the males (M) and females (F) from test 1 (T1) and test 2 (T2), as well as the results of the reliability analyses.

Subject	T1 (RPE_GET_)	T2 (RPE_GET_)	T1 (GET _%RPE peak_)	T2 (GET _%RPE peak_)	T1 (RPE_RCP_)	T2 (RPE_RCP_)	T1 (RCP _%RPE peak_)	T2 (RCP _%RPE peak_)
M1	15	17	71	65	15	17	86	92
M2	13	14	53	61	13	14	81	89
M3	19	17	64	60	19	17	93	86
M4	17	18	72	73	17	18	83	88
M5	17	18	79	78	17	18	85	88
M6	16	17	69	68	16	17	81	87
M7	15	16	59	58	15	16	81	82
M8	16	17	74	78	16	17	83	91
M9	18	17	66	64	18	17	88	83
**Mean ± SD**	**13 ± 2**	**13 ± 2**	**67 ± 8**	**67 ± 8**	**16 ± 2**	**17 ± 1**	**85 ± 4**	**87 ± 3**
**ICC**	**0.886**		**0.869**		**0.651**		**−0.093**	
**[95% CI]**	**[0.604–0.973]**		**[0.516–0.969]**		**[0.094–0.906]**		**[−0.572–0.535]**	
**SEM**	**1**		**3%**		**1**		**4%**	
**MD**	**2**		**8%**		**2**		**11%**	
**CV**	**5.46%**		**4.38%**		**5.31%**		**4.50%**	
**Mean ± SD**	**13 ± 2**		**67 ± 8%**		**16 ± 1**		**86 ± 4%**	
**Bias (SD)**	**0.01 (0.71)**		**0.23 (3.97)**		**−0.55 (1.01)**		**−2.60 (5.20)**	
**[LOA]**	**[−1.39–1.40]**		**[−7.55–8.01]**		**[−2.53–1.42]**		**[12.80–7.60]**	
F10	12	14	68	72	15	17	84	92
F11	12	11	62	55	15	17	81	89
F12	14	14	68	71	18	18	92	91
F13	12	12	64	62	18	17	97	88
F14	14	13	74	66	18	18	93	91
F15	11	13	58	70	18	19	97	99
F16	15	16	77	78	19	18	93	92
F17	13	14	66	72	17	18	91	94
F18	13	15	65	78	18	18	92	95
**Mean ± SD**	**13 ± 1**	**13 ± 2**	**67 ± 6**	**69 ± 7**	**17 ± 1**	**18 ± 1**	**91 ± 5**	**92 ± 3**
**ICC**	**0.573**		**0.382**		**0.467**		**0.312**	
**[95% CI]**	**[−0.008–0.880]**		**[−0.303–0.815]**		**[−0.176–0.844]**		**[−0.432–0.792]**	
**SEM**	**1**		**5%**		**1**		**4%**	
**MD**	**2**		**15%**		**2**		**10%**	
**CV**	**6.60%**		**7.7%**		**4.53%**		**4.08%**	
**Mean ± SD**	**13 ± 1**		**68 ± 7**		**18 ± 1**		92 ± 4% *	
**Bias (SD)**	**−0.63 (1.11)**		**−2.52 (6.91)**		**−0.47 (0.94)**		**−1.42 (5.03)**	
**[LOA]**	**[−2.81–1.54]**		**[−16.06–11.02]**		**[−2.32–1.38]**		**[−11.28–8.43]**	

Note: ICC = intraclass correlation coefficient, CI = confidence interval, SEM = standard error of the measurement, MD = minimal difference to be considered real, CV = coefficient of variation, CE = Constant Error, LOA = limits of agreement. * Indicates a significantly greater normalized RCP of RPE (% of RPE_peak_) for the females compared to males.

## Data Availability

The original contributions presented in this study are included in the article. Further inquiries can be directed to the corresponding author. Individual subject data are reported in Table 2, Table 3, Table 4, Table 5 and Table 6 throughout this manuscript.

## Abbreviations

The following abbreviations are used in this manuscript:

**GET**	gas exchange threshold
**RCP**	respiratory compensation point
**$\dot{V}$O_2_**	volume of oxygen consumption
**GXT**	graded exercise test
**PO**	power output
**HR**	heart rate
**RPE**	rating of perceived exertion
**ICC**	intraclass correlation coefficient
**MD**	minimal difference
**SEM**	standard error of the measurement
**CV**	coefficient of variation
**% $\dot{V}$O_2max_**	percentage of maximum oxygen consumption
**VT_1_**	first ventilatory threshold
**VT_2_**	second ventilatory threshold
**CP**	critical power
**$\dot{V}$CO_2_**	volume of carbon dioxide expiration
**$\dot{V}$E**	ventilatory equivalents
**PPO**	peak power output
**$\dot{V}$O_2peak_**	peak oxygen consumption
**HR_peak_**	heart rate peak
**RPE_peak_**	rating of perceived exertion peak
**PCO_2_**	partial pressure of carbon dioxide
**PO_GET_**	power output at the gas exchange threshold
**PO_RCP_**	power output at the respiratory compensation point
**HR_GET_**	heart rate at the gas exchange threshold
**HR_RCP_**	heart rate at the respiratory compensation point
**RPE_GET_**	rating of perceived exertion at the gas exchange threshold
**RPE_RCP_**	rating of perceived exertion at the respiratory compensation point
**HR _NORM GET_**	heart rate normalized to the gas exchange threshold
**HR _NORMM RCP_**	heart rate normalized to the respiratory compensation point
**RPE _NORM GET_**	rating of perceived exertion normalized to the gas exchange threshold
**RPE _NORM GET_**	rating of perceived exertion normalized to the respiratory compensation point
**T1**	test one
**T2**	test two
**GET _%PPO_**	gas exchange threshold expressed as a percentage of peak power output
**RCP _%PPO_**	respiratory compensation point expressed as a percentage of peak power output
**CI**	confidence interval
**SD**	standard deviation
**% of $\dot{V}$O_2peak_**	percentage of peak oxygen consumption
**GET % HR_peak_**	gas exchange threshold expressed as a percentage of peak heart rate
**RCP % HR_peak_**	respiratory compensation point expressed as a percentage of peak heart rate
**GET % RPE_peak_**	gas exchange threshold expressed as a percentage of peak rating of perceived exertion
**RCP % RPE_peak_**	respiratory compensation point expressed as a percentage of peak rating of perceived exertion
